# Vascular, glial, and lymphatic immune gateways of the central nervous system

**DOI:** 10.1007/s00401-016-1606-5

**Published:** 2016-08-13

**Authors:** Britta Engelhardt, Roxana O. Carare, Ingo Bechmann, Alexander Flügel, Jon D. Laman, Roy O. Weller

**Affiliations:** 1 Theodor Kocher Institute, University of Bern, 3012 Bern, Switzerland; 2 Faculty of Medicine, University of Southampton, Southampton, UK; 3 Institute of Anatomy, University of Leipzig, Leipzig, Germany; 4 Institute of Neuroimmunology and Institute for Multiple Sclerosis Research, University Medical Centre Göttingen, 37073 Göttingen, Germany; 5 Department of Neuroscience, University Medical Center Groningen (UMCG), University of Groningen, 9713 AV Groningen, The Netherlands; 6 Neuropathology, Mailpoint 813, Level E, South Block, Southampton University Hospital, Southampton, SO16 6YD UK

**Keywords:** CNS, CSF, Interstitial fluid, Immune privilege, Lymphatic drainage, Blood–brain barrier, Antigen-presenting cells, Dendritic cells, Glia limitans: multiple sclerosis, Alzheimer’s disease

## Abstract

Immune privilege of the central nervous system (CNS) has been ascribed to the presence of a blood–brain barrier and the lack of lymphatic vessels within the CNS parenchyma. However, immune reactions occur within the CNS and it is clear that the CNS has a unique relationship with the immune system. Recent developments in high-resolution imaging techniques have prompted a reassessment of the relationships between the CNS and the immune system. This review will take these developments into account in describing our present understanding of the anatomical connections of the CNS fluid drainage pathways towards regional lymph nodes and our current concept of immune cell trafficking into the CNS during immunosurveillance and neuroinflammation. Cerebrospinal fluid (CSF) and interstitial fluid are the two major components that drain from the CNS to regional lymph nodes. CSF drains via lymphatic vessels and appears to carry antigen-presenting cells. Interstitial fluid from the CNS parenchyma, on the other hand, drains to lymph nodes via narrow and restricted basement membrane pathways within the walls of cerebral capillaries and arteries that do not allow traffic of antigen-presenting cells. Lymphocytes targeting the CNS enter by a two-step process entailing receptor-mediated crossing of vascular endothelium and enzyme-mediated penetration of the glia limitans that covers the CNS. The contribution of the pathways into and out of the CNS as initiators or contributors to neurological disorders, such as multiple sclerosis and Alzheimer’s disease, will be discussed. Furthermore, we propose a clear nomenclature allowing improved precision when describing the CNS-specific communication pathways with the immune system.

## Introduction

The central nervous system (CNS), comprising the brain, spinal cord, and neural parts of the eye, has a unique relationship with the immune system that has been referred to as immune privilege. Support for the concept of immune privilege for the CNS arose from experiments by Shirai nearly 100 years ago in which foreign homologous tissues were grafted to the brain and survived for prolonged periods; the concept was further emphasised by Medawar in 1948 [29, 86]. Nevertheless, immunological reactions do occur within the CNS particularly in association with infections by microorganisms and with diseases, such as multiple sclerosis (MS), that have an autoimmune component. As a sequel to the grafting experiments, it was shown that if the same allografts were subsequently grafted on to the skin, allografts in the brain were rapidly rejected [29, 86]. It appears, therefore, that immunization in peripheral tissues precipitates immunological rejection of foreign tissue in the CNS. The description of immune privileged sites as those in which: “grafts transplanted to them are in some way partially or fully exempted from the normal rigours imposed by their histocompatibility status” is a definition of a state of relative tolerance [12, 45]. One further point that has been emphasised is that immune privilege does not apply to the meninges and CSF spaces [45]. If foreign tissue grafted into the brain enters the ventricles, it is rejected [45, 85].

Except for penetrating injuries of the brain or spinal cord, microorganisms do not enter the CNS unless they have passed through peripheral tissues, such as lung, gut, skin, or nasal mucosa. Thus, T lymphocyte-mediated immune responses, such as those involved in the rejection of skin allografts, in the elimination of microorganisms and in autoimmune disorders, may only occur in the CNS in individuals that have been initially immunised by antigen in peripheral tissues.
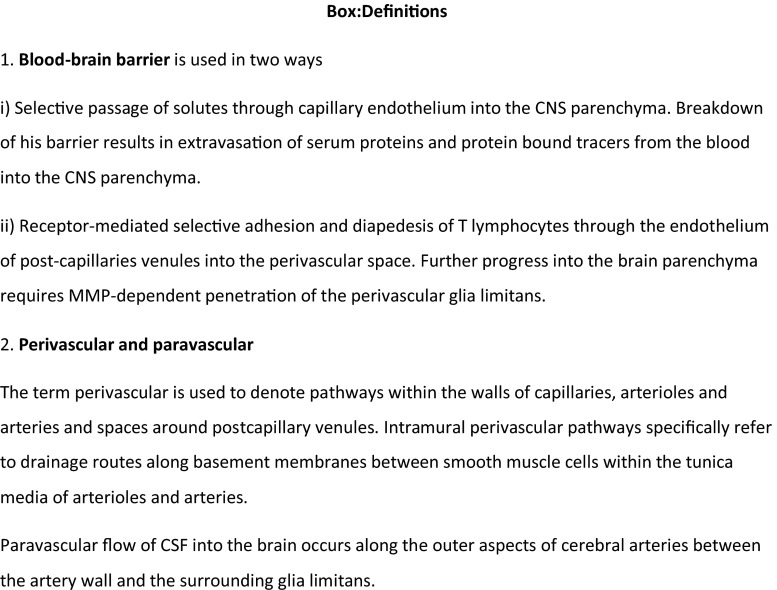


Immunological reactions comprise two major components: an innate response and an adaptive response. For the CNS, the innate response within the parenchyma itself is mediated by yolk sac-derived myeloid cells differentiating into resident microglia. When present, MHC class I and II molecules on the surfaces of cells constantly present antigen. In the absence of an infection, this is self-antigen and thus contributes to tolerance. Expression of MHC class I and II molecules on microglial cells has been shown to be suppressed by the electrical activity of CNS neurons [94]. Disturbance of this activity during neurological disorders combined with cytokine activation of microglial cells leads to expression of MHC class I and class II molecules on their surfaces and subsequent presentation of antigen to receptive T lymphocytes [13, 70, 80]. The adaptive response depends upon the presentation of antigen to lymphocytes by antigen-presenting cells (APC). In addition, the leptomeningeal and perivascular compartments of the CNS harbour professional APC, such as macrophages and dendritic cells (DC), that, without activating signals, constitutively express MHC class I and class II molecules and constantly present self-antigens and thus may promote T-cell anergy. However, once activated, these APC have the potential to become the principal activators of T-cell responses in the CNS.

In tissues other than the CNS, activated APC migrate within well-defined lymphatic vessels to regional lymph nodes that present an environment specialised for antigen presentation in terms of anatomical and cell composition. Following the presentation of antigen, T and B lymphocytes proliferate to produce activated effector T cells and antibodies that return to the blood and to their target organs. Activated B cells also pass into the blood stream and then to bone marrow and may target tissues in which they can differentiate into plasma cells the CNS.

It has been proposed that immune privilege in the CNS is a result of two major factors: (a) the blood–brain barrier (BBB) (blood–CNS barrier) restricting entry of immune cells into the CNS and (b) the absence of the conventional lymphatics in the CNS. Both these factors will be discussed in the present review.

**Fig. 1:**
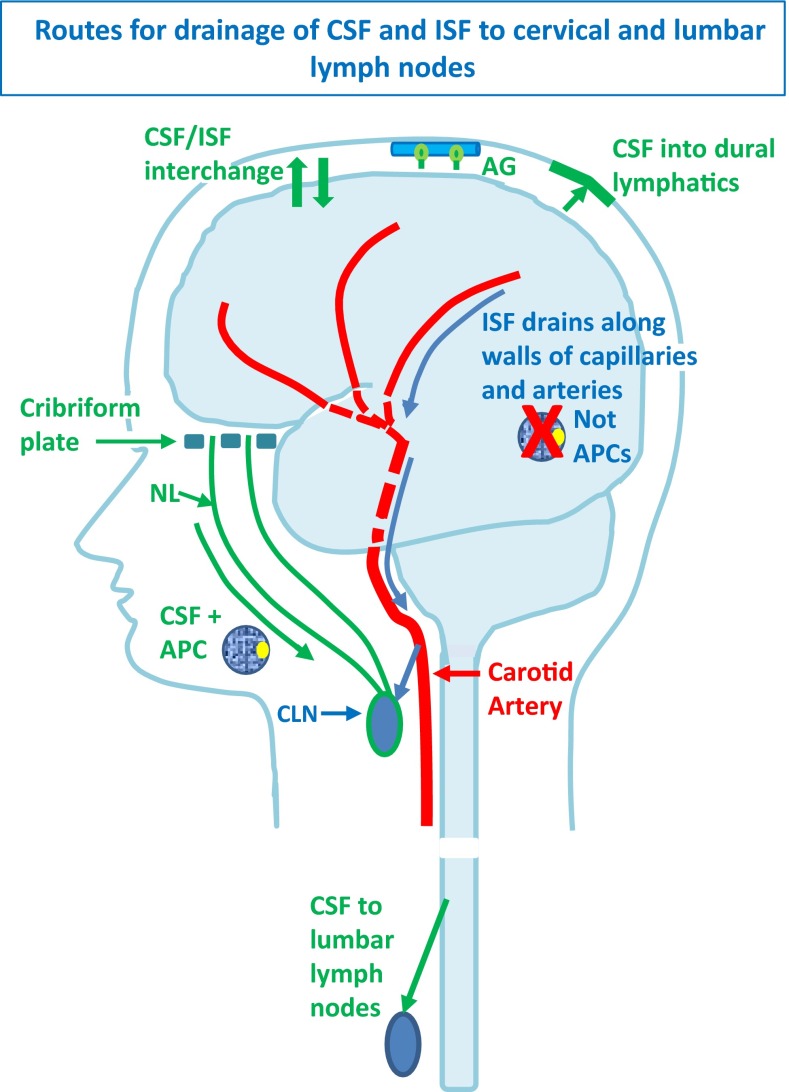
Drainage pathways for CSF and interstitial fluid (ISF) to cervical lymph nodes. CSF and ISF drain to lymph nodes by different and distinct pathways. In humans, CSF drains into the blood of venous sinuses through well-developed arachnoid villi and granulations (AG). Lymphatic drainage of CSF occurs via nasal and dural lymphatics and along cranial and spinal nerve roots (outlined in *green*). Channels that pass from the subarachnoid space through the cribriform plate allow passage of CSF (*green line*) T cells and antigen-presenting cells (APC) into nasal lymphatics (NL) and cervical lymph nodes (CLN). CSF from the lumbar subarachnoid space drains to lumbar lymph nodes. ISF from the brain parenchyma drains along basement membranes in the walls of cerebral capillaries and arteries (*blue arrows*) to cervical lymph nodes adjacent to the internal carotid artery just below the base of the skull. This narrow intramural perivascular drainage pathway does not allow the traffic of APC. There is interchange between CSF and ISF (convective influx/glymphatic system), as CSF enters the surface of the brain alongside penetrating arteries

There are two major extracellular fluids within the cranial and spinal cavities associated with the CNS that could carry antigen or APC to regional lymph nodes, namely: cerebrospinal fluid (CSF) in the ventricles and subarachnoid spaces and interstitial fluid (ISF) in the extracellular spaces of the brain and spinal cord parenchyma. As will be outlined in this review, both CSF and ISF drain to cervical and lumbar lymph nodes, but there are significant differences between their drainage pathways (Fig. 1). CSF drains from the subarachnoid space into lymphatic vessels in the nasal mucosa, in the dura mater, and into lymphatics associated with the sheaths of cranial and spinal nerve roots [11, 33, 69, 81]. The nasal and dural routes appear to allow the traffic of APC to lymph nodes [66, 81]; CSF compartments, (the ventricles and subarachnoid space), do not exhibit the same immune privilege as the parenchyma of the CNS. In contrast to CSF, ISF drains from the brain parenchyma to cervical lymph nodes along very narrow, restricted pathways that comprise 100–150 nm-thick basement membranes in the walls of cerebral capillaries, arterioles, and arteries [24, 26] (Fig. 1). Such intramural perivascular basement membrane pathways are not large enough to allow the traffic of APC to regional lymph nodes and this may be a key factor in inducing immune privilege in the parenchyma of the CNS [24, 26].

In this review, we compare the connections between the CNS and the immune system with those of peripheral organs. The anatomical pathways and the physiology of lymphatic drainage of the CNS and how immune cells enter the CNS through the BBB are discussed in some detail. We examine the possible mechanisms involved in immunological diseases of the CNS, how lymphatic drainage pathways are involved in the aetiology of Alzheimer’s disease and we also revisit the concept of immune privilege.

## Lymphatic drainage of systemic organs other than the CNS

**Fig. 2:**
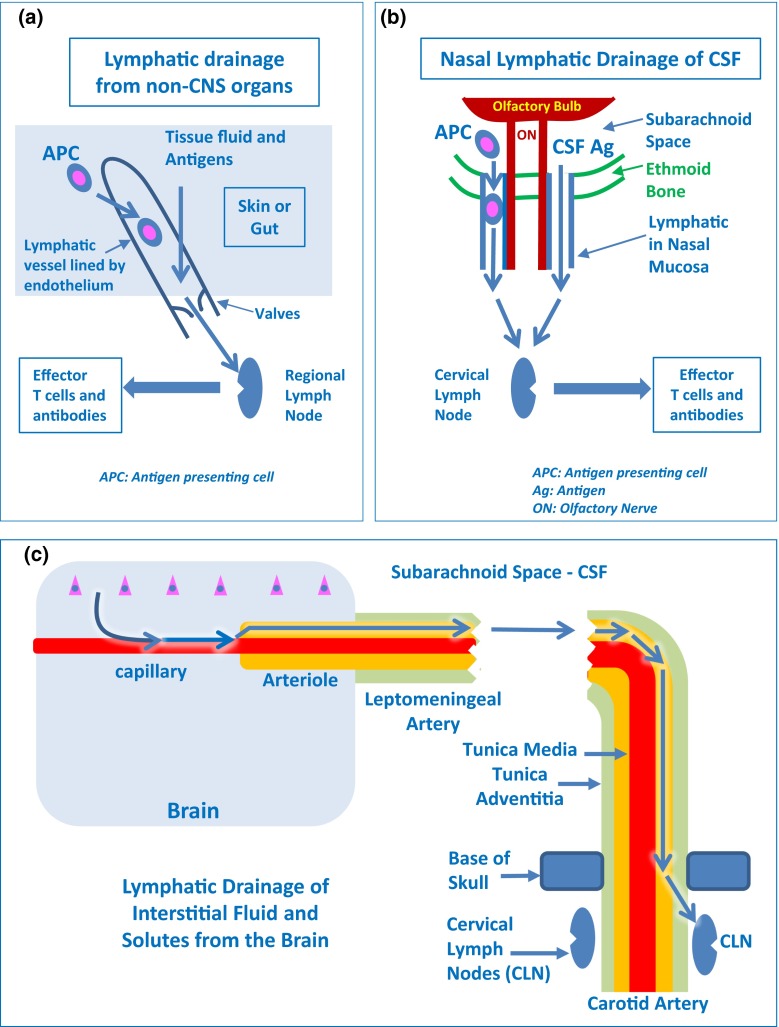
Pathways for lymphatic drainage of peripheral organs, CSF and cerebral interstitial fluid. **a** Tissue fluid from skin, gut, and other organs drains into blind-ended lymphatic vessels lined by endothelium. Antigen-presenting cells (APC) also drain by this route to regional lymph nodes. Valves are shown in the lymphatic vessel. **b** CSF and antigen-presenting cells (APC) pass from the subarachnoid space through channels in the ethmoid bone, alongside olfactory nerves (ON), to enter lymphatics in the nasal mucosa and drain to cervical lymph nodes. CSF also drains via dural lymphatics towards the deep cervical lymph nodes (see text) **c** Interstitial fluid (ISF) from the brain parenchyma enters basement membranes in the walls of capillaries and then basement membranes between smooth muscle cells in the tunica media of cerebral arterioles and arteries. The route of intramural perivascular drainage for ISF is indicated by* blue arrows* that track along the walls of intracranial arteries to cervical lymph nodes (CLN) related to the internal carotid artery at the base of the skull

Lymphatic drainage into regional lymph nodes is normally involved in raising adaptive immunological responses in tissues. ISF continuously extravasates from blood vessels and drains from tissues to lymph nodes. In most tissues of the body, except for the brain, spinal cord and parts of the eye, specialized lymphatic vessels begin as blind-ended capillaries and allow the free uptake of excess protein-rich ISF via discontinuous button-like junctions between lymphatic endothelial cells [6] (Fig. 2a). Once within the lymphatic vessels, tissue fluid is referred to as lymph. Lymph moves from the lymphatic capillary bed into pre-collector vessels that connect to collecting lymphatic vessels (afferent lymphatics), as they drain lymph directly into the regional lymph nodes. While contractility of smooth muscle cells surrounding collecting lymphatic vessels and arterial pulsations ensure the forward movement of the lymph, valves of lymphatic endothelium prevent backflow of lymph. The lymph drains out of lymph nodes via efferent lymphatic vessels. In general, lymph nodes are arranged in series and the lymph eventually reaches two large lymphatic vessels, the thoracic duct and the right lymphatic duct that empty the lymph into the superior vena cava. Thus, the fluid is eventually transported back into the blood circulation and ensures tissue fluid homeostasis.

Lymphatic vessels have important functions for immune surveillance, as they transport antigens and activated APC, such as macrophages and DCs, from the peripheral tissues into the lymph nodes allowing adaptive immune responses to be mounted. Activated effector T and B cells and humoral factors, such as antibodies, are then delivered by lymphatic vessels into the blood stream.

When DCs residing in tissues take up foreign antigens, they become activated, a process that includes a loss of their tissue adhesive characteristics and upregulation of the chemokine receptor CCR7. These two factors induce the migration of DCs into lymphatic vessels by engaging the CCR7 ligand CCL21 specifically expressed by lymphatic endothelial cells. DCs first crawl along the lymphatic endothelium using specific adhesive interactions, e.g., the cytokine CCL21, before they detach and are passively transported to the regional lymph nodes in the larger calibre lymphatic vessels [97, 115]. Once they have arrived in the lymph node, DCs activate antigen-specific T cells that in turn proliferate and reach the blood stream via the efferent lymphatic vessels. The activation of B cells is mediated by the binding of soluble antigens to the B-cell receptors; in the case of protein antigens, they are internalized by DCs and presented to CD4^+^ T cells which in turn activate the B cells. Activated B cells and antibodies also reach the blood stream via efferent lymphatic vessels.

Interestingly, mouse models have provided evidence that some milieux in the body imprint immune cells to develop tissue-specific-trafficking programs. Environmental cues from food (e.g., vitamin A) and sunlight (UV induced vitamin D3) are metabolized by DCs which allows them to imprint tissue-specific homing patterns in activated effector lymphocytes during the process of antigen presentation [125]. Effector T cells produced in lymph nodes that drain the skin express the chemokine receptors CCR4 and CCR10 and the cutaneous lymphocyte antigen, while effector T cells produced in lymph nodes that drain the gut express CCR9 and α4β7 integrin. This allows the different effector T-cell subsets to specifically home to the skin or to the gut once they are released back into the blood stream. Specific homing is achieved by the T cells engaging tissue-specific vascular ligands (CCL27, CCL17, and E-selectin) (for skin) or CCL25 and MAdCAM-1 (mucosal cell adhesion molecule −1) (for gut); these ligands are upregulated on the inflamed vascular endothelial cells in the skin or gut microvessels. Trafficking of lymphocytes to selected tissues provides a mechanism for segregating specialized adaptive immune responses to unique immune microenvironments. At least for the skin and the gut, DCs thus play a central role in this process, as, in addition to presenting antigens, they metabolize vitamins and respond to local tissue cues, including cytokines that they export to the regional lymph nodes.

## Lymphatic drainage of the CNS

Of the two extracellular tissue fluids associated with the CNS, CSF is mainly located in the ventricles and subarachnoid spaces and has a total volume in humans of 140 mL [19]. The other fluid is ISF in the extracellular spaces of the brain and spinal cord parenchyma and amounts to 280 mL in humans [19]. Both CSF and ISF drain to lymph nodes and are involved in immunological reactions within the CNS [34, 74, 106].

### Lymphatic drainage of cerebrospinal fluid

CSF is mainly produced by the choroid plexuses in the cerebral ventricular system at the rate of 350 µL/min in humans [35]. A proportion of CSF may be derived from ISF [64]. Passing from the ventricular system into the subarachnoid spaces, CSF in humans then drains partly into the blood via arachnoid villi and granulations in the walls of venous sinuses. A further proportion of CSF drains to regional lymph nodes via nasal lymphatics and dural lymphatics and via lymphatic vessels associated with cranial and spinal nerve roots [11, 33, 65, 69, 81] (Fig. 1). It is estimated that at least 50 % of CSF drains into lymphatics in some mammals, [33] but the proportion in humans is still unknown [65].

The most prominent pathway for the lymphatic drainage of CSF is via channels that connect the subarachnoid space with lymphatic vessels in the nasal mucosa via the cribriform plate of the ethmoid bone (Fig. 2b). This pathway was demonstrated in humans in 1912 [148] and has more recently been confirmed in more detail in rats and other mammals, including humans [65, 69]. CSF from the spinal subarachnoid space drains to lumbar lymph nodes [69].

Two recent articles elegantly demonstrated functional lymphatic vessels in the dura mater of the mouse situated bilaterally along the superior sagittal sinus and draining through the cribriform plate into the nasal mucosa [11, 81]. Lymph vessels of the dura mater were probably described first by Mascagni in 1787 in his study “De lymphaticis profundis capitis et colli” [21], and since then, functional drainage by this route has been demonstrated by Schwalbe et al. [121], Andres et al. [9], Cserr and Knopf [34], Kida et al. [69], and more recently by Aspelund et al. [11] and by Louveau et al. [81]. Although it has been suggested that lymphatic vessels in the dura drain fluid, solutes, and cells from the brain parenchyma, no such drainage has been demonstrated and the anatomical pathways for such drainage have not been demonstrated. It is more likely that dural lymphatics are one of the pathways for the drainage of CSF.

The capacity of the lymphatic drainage pathways for the CSF is reflected in experiments using particulate matter, such as Indian ink and Microfil [65, 69]. Several studies have also demonstrated efflux of T cells and APC from the CSF into deep cervical lymph nodes [47, 50, 51, 66, 101]. Moreover, myelin and axonal epitopes have been found in deep cervical lymph nodes after axonal injury and (autoimmune) demyelination [36, 40, 79, 93]. Although it can be emphasised that APC may drain with CSF to lymph nodes by various routes, the migration of APC from brain parenchyma with ISF along narrow intramural periarterial pathways is highly unlikely (see section on “Intramural perivascular drainage of ISF and solutes from the brain parenchyma”).

Current challenges thus include the clear identification of the anatomical routes by which APC traffic from the CSF and CNS parenchyma to deep cervical lymph nodes, and the unravelling of the role of antigen-presentation in neuroinflammatory and neurodegenerative diseases.

### Drainage of interstitial fluid from the CNS parenchyma to regional lymph nodes

**Fig. 3:**
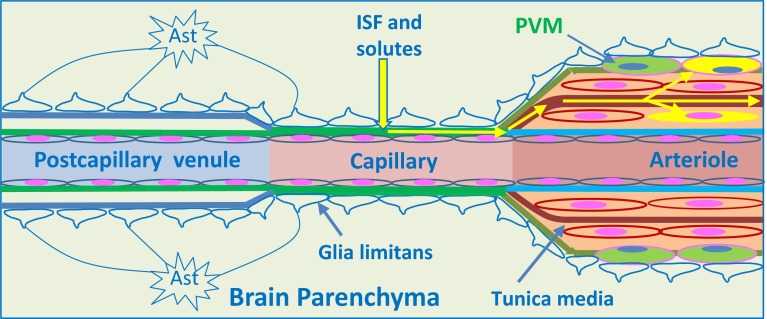
Intramural perivascular drainage of interstitial fluid (ISF) out of the brain parenchyma. As an arteriole loses its tunica media, it becomes a capillary and then a post-capillary venule. Astrocyte end feet closely invests the surface of capillaries and form the glia limitans. Capillary walls are the site of the blood–brain barrier (BBB) for solutes; the glial and endothelial components of the basement membrane (*green*) are fused. The wall of the post-capillary venule is the BBB for lymphocytes and other inflammatory cells to cross from blood to CNS (see Fig. 6); the glial (*blue*) and endothelial (*green*) basement membranes are not fused. Interstitial fluid (ISF) and solutes drain from the extracellular spaces in the brain parenchyma through gaps between astrocyte end feet (*yellow arrow*) to enter bulk flow channels in basement membranes of cerebral capillaries. From there, ISF drains into basement membranes between smooth muscle cells in the tunica media of arterioles and arteries (*yellow arrows*): this is the intramural perivascular drainage pathway. Tracers following this pathway are taken up by smooth muscle cells in the tunica media and by perivascular macrophages (PVM) on the outer aspects of arterioles and arteries. Neither the endothelial basement membrane of arterioles and arteries (*light blue*) nor the outer basement membranes of the artery wall (*green*) are involved in the intramural perivascular drainage of ISF from the CNS

**Fig. 4:**
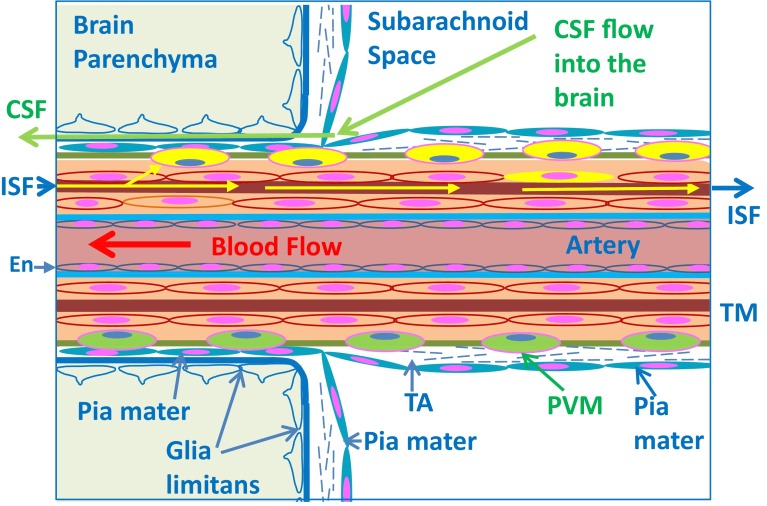
Artery at the surface of the cerebral cortex. This figure shows the relationship of the artery with the subarachnoid space, the pathway for entry of CSF into the brain, and the intramural perivascular pathway for the drainage of interstitial fluid (ISF) out of the brain. Blood flows along the lumen of the artery into the brain. The arterial endothelium (En) is separated from the tunica media (TM) by a basement membrane (*light blue*), that is not involved in ISF transport, and by the extracellular matrix of the tunica intima. ISF and solutes flow out of the brain along basement membranes (*brown*) between smooth muscle cells in the tunica media. As tracers in the ISF flow along this pathway (*yellow arrows*), they are taken up by perivascular macrophages (PVM—*coloured yellow*) and by smooth muscle cells in the tunica media. The pia mater forms a continuous layer of (*blue coloured*) cells; it coats the wall of the artery in the subarachnoid space, fuses with the pia mater on the surface of the brain, and extends as a layer of pia closely applied to the artery, as it enters the brain. CSF enters the brain from the subarachnoid space (the convective influx/glymphatic pathway) along the basement membrane (*dark blue*) that is shared by the pia mater and the astrocytes of the glia limitans. In the normal cerebral cortex, there is no perivascular (Virchow–Robin) space around arteries, as they enter the brain. Tunica adventitia (TA) coats the leptomeningeal artery in the subarachnoid space, and perivascular macrophages (PVM) are aligned along the outer parts of the artery wall

Experimental studies have shown that soluble tracers injected in minute amounts (0.5 μL) into the parenchyma of grey matter areas in the mouse brain initially diffuse through the extracellular spaces (ECS) and then rapidly enter the basement membranes of cerebral capillaries and drain directly via basement membranes in the tunica media of arterioles and arteries out of the brain to cervical lymph nodes [24, 26, 33] (Figs. 1, 2c, 3, 4). This intramural perivascular drainage route is outlined in the human brain by deposits of amyloid β (Aβ) in basement membranes in cerebral amyloid angiopathy (CAA) associated with age and with Alzheimer’s disease [25, 141]. Injection of larger volumes (2 μL) of tracer into the striatum of the mouse brain results in the passage of tracer into CSF in the ventricles [15].

### Origins of ISF

Water generated as a result of oxidation of glucose to CO_2_ could provide a 10 % contribution to the total volume of ISF, but recently, it has been proposed that a large fraction of ISF is derived from the blood, passing through the capillary endothelium and driven by Na, K ATPase with water following passively [2]. In addition to water, ISF consists of tissue metabolites and secreted proteins. The rate of bulk flow of ISF is estimated to be 0.1–0.3 µL min^−1^ g^−1^ in the rat brain [2]. Tracer experiments and mathematical models show that ISF is eliminated from the brain by bulk flow along white matter fibre tracts and along perivascular pathways [32, 119].

Electron microscopy has shown that very narrow gaps separate the cells of the CNS, particularly in the grey matter [58, 91]. The gaps are interconnected and are filled with ISF; they represent the extracellular spaces (ECS) and are in direct continuity with the basement membranes of capillaries [89]. The most significant exchange between blood and CNS occurs at the capillary level. Cerebral capillary endothelial cells are connected by highly complex and continuous tight junctions and form the BBB. While crossing the BBB requires specific transport systems, once beyond the BBB molecules diffuse over short distances of 8–25 μm to the surrounding cells comprising the CNS. The movement of water mixed with the movement of soluble metabolites results in the flow of ISF [2, 3]. ISF drains from the ECS of the CNS parenchyma by entering capillary basement membranes and then draining along the basement membranes of cerebral arteries by bulk flow, as indicated by physiological tracer experiments [24]. Bulk flow of ISF also occurs along the white matter fibre tracts [2]. Functionally, the ECS provides a pathway for the diffusion and exchange of ions and molecules between cells; the movement of ISF through the ECS surrounding the cells of nervous system depends on diffusion [96, 130]. Diffusion through the ECS and bulk flow of ISF along perivascular drainage routes changes with age and in Alzheimer’s disease [54, 87].

### Drainage of ISF along intramural perivascular pathways

Experimental studies in the 1980s by Cserr et al. first identified the perivascular route of drainage for ISF from the brain to cervical lymph nodes. These authors injected minute amounts of radioiodinated serum albumin tracer into various regions of the rat brain and showed that the tracer drained to cervical lymph nodes along the walls of cerebral arteries [33]. The speed of drainage to lymph nodes was comparable to that of lymphatic drainage elsewhere in the body. Only 10–15 % of tracer injected into the caudate nucleus or internal capsule passed into the CSF, although this figure was higher for tracer injected into the midbrain [131].

In this review, we will examine how the work of Cserr et al. has been expanded by the use of fluorescent tracers and confocal microscopy and how the perivascular drainage of ISF is relevant to neuroimmunology and to the pathogenesis of Alzheimer’s disease. Before examining the evidence for intramural perivascular clearance of ISF and solutes from the brain parenchyma, we will examine the structure of cerebral blood vessels and the vascular basement membranes that form the pathways for perivascular drainage. Individually, blood vessels in the mouse brain have a similar basic structure to those in the human brain [76].

### Capillaries

Capillary walls in the human and mouse brain are formed by an endothelium and a basement membrane, separating it from the brain ECS (Fig. 3) [91, 110]. While the junctions between adjacent endothelial cells form unique zonulae occludentes that inhibit paracellular diffusion of solutes, the high number of pericytes embedded in brain capillary basement membranes inhibits transcellular vesicular activity and thus transcellular diffusion of solutes [17]. Fibrous astrocytes form end feet that completely surround the capillary surface: 40–100 nm separate the astrocyte end feet from the endothelium and this space is occupied by basement membrane [38]. The layer of basement membrane produced by endothelial cells is distinct in its molecular structure from the basement membrane of the glia limitans produced by astrocytes, but the two basement membranes are fused. The capillary basement membrane is in direct communication with the brain extracellular spaces and encircles pericytes [142] that are responsible for the regulation of capillary diameter and of cerebral blood flow in response to neural activity [16].

### Arteries and arterioles

A normal cerebral artery on the surface of the brain (leptomeningeal artery) has an endothelium separated from layers of smooth muscle cells by basement membrane, an intimal layer of extracellular matrix, and, frequently, an internal elastic lamina [138, 144]. As an artery enters the cerebral cortex, it loses its internal elastic lamina and its tunica adventitia to become an arteriole (Fig. 4). Basement membranes are interposed between individual smooth muscle cells, and between the smooth muscle layers of the artery wall and its outer leptomeningeal sheath. The parenchymal basement membrane of the glia limitans separates the astrocyte end feet from the periarterial leptomeningeal sheath (Fig. 4). Smooth muscle cells within the artery wall possess contractile properties of fundamental importance for the regulation of cerebral blood flow. They are able to respond to mechanical (pressure), electrical, chemical, and hormonal stimuli [72]. The adhesion of the vascular smooth muscle cells to their basement membranes is essential for maintaining the integrity of the arterial wall and for recognizing and integrating the high variety of signals that modulate the cell function [126].

Studies of human brain show that leptomeningeal cells, identified by the presence of desmosomes and small nexus junctions, are reflected from the surface of the brain and spinal cord to coat arteries and veins in the SAS, thus separating CSF in the subarachnoid space from the CNS and perivascular compartments [95, 137] (Fig. 4). Furthermore, leptomeningeal cells form a perivascular sheath around arteries, as they enter the brain. Periarterial compartments in the brain are separated from the subarachnoid space by pia mater (Fig. 4), but are in direct continuity with periarterial compartments of leptomeningeal vessels. Such compartments continue as basement membranes in the tunica media and as a layer of perivascular (adventitial) connective tissue coating arteries, as they pass through the base of the skull. Venous perivascular spaces communicate with the subpial space, as veins tend to lack a complete perivascular coat of leptomeningeal cells [146].

The pia mater prevents particles and erythrocytes [60] and, probably, neurotransmitters [41] in the CSF from entering perivascular compartments of the brain, but the exact degree of selective permeability to solutes of the pia mater and underlying glia limitans is not known. There are regional differences in the structure of arteries within the brain as shown by the enlarged perivascular spaces that develop with age around arteries in the basal ganglia [109] and white matter [140]; the perivascular spaces are enclosed by two layers of leptomeninges in these regions of the brain [109]. Arteries in the cortex, on the other hand, have only one layer of encompassing leptomeningeal cells and do not have perivascular space [146] (Fig. 4).

Vascular basement membranes are 100–150 nm-thick laminar matrices produced by endothelial, astroglia, smooth muscle cells, and pericytes [144] (Figs. 3, 4). In addition to providing a potential pathway for the clearance of solutes out of the brain, the extracellular matrix, including the vascular basement membrane, determines the mechanical properties of the vessel wall and controls the migration and differentiation of vascular cells. In the brain, basement membranes have been reported to be secreted by meningeal cells and contribute to the migration and final positioning of neurons and to the differentiation of the laminar cortical pattern during development [89]. Basement membranes are composed mainly of collagen type IV, laminins, nidogens, fibronectin, and heparan sulphate proteoglycans. The basement membrane is a dynamic complex, capable of remodelling itself. In vitro, type IV collagen, laminin, and fibronectin are capable of assembly into a protein network resembling basement membranes and are interdependent in the formation of the basement membranes [145].

### Intramural perivascular drainage of ISF and solutes from the brain parenchyma

To define the anatomical pathway for the bulk flow drainage of ISF and solutes from the brain parenchyma, minute quantities (0.5 μL) of fluorescent dextran 3KDa or ovalbumin 49KDa were injected into the grey matter of the caudate putamen in the centre of the mouse cerebral hemisphere [24]. By 5 min after injection, tracers had spread diffusely through the ECS, but tracer was also present in the walls of blood vessels. Confocal microscopy showed that tracers were co-localized with laminin in the basement membranes of capillaries and in the basement membranes in the tunica media of arteries at time intervals of 5–15 min after intracerebral injections into the caudate putamen [24] (Fig. 3). The location of the tracers in artery walls was very specific, as tracer was only in the basement membranes between the smooth muscle cells in the tunica media and not in the endothelial basement membranes or in the outer basement membrane encompassing the artery wall [24]. This defined the drainage route for ISF as an intramural perivascular pathway. In the normal brain, there are no actual “perivascular spaces” around arteries, as they enter the cerebral cortex (Fig. 4) [90, 137, 146]. The drainage route for ISF and solutes is along pathways within the tunica media of arteries and not along perivascular spaces (Fig. 2).

Some of the tracer, particularly 3KDa dextran, was taken up by a few smooth muscle cells in the tunica media and by perivascular macrophages on the outer aspects of the artery walls (Fig. 3). By 30 min after injection, the tracers had disappeared from the ECS of the brain and from the basement membranes in the walls of capillaries and arteries, but tracers remained in the perivascular macrophages [24]. By 24 h after injection, the route taken by the tracers on their passage out of the brain was outlined by perivascular macrophages containing tracer and situated adjacent to intracerebral arteries and around leptomeningeal arteries on the surface of the brain [24].

The intramural perivascular drainage route is fast and specific to solutes and does not allow direct migration of APC from the CNS parenchyma to lymph nodes. When particles in the range of 15 nm–1 μm are injected into grey matter in the brain, they do not drain along intramural basement membranes, but track along the outside of arteries and separate the glia limitans from the vessel walls [24, 147]. It was concluded from these studies that it was very unlikely that, due to their size, APC would be able to track along intramural arterial basement membranes from the brain to cervical lymph nodes [24].

Studies using radioiodinated serum albumin as a tracer suggest that once ISF and solutes have left the brain, they drain along the tunica media and the tunica adventitia of the major cerebral arteries, through the base of the skull to deep cervical lymph nodes [131] (Fig. 2c). The high levels of radioactive tracer and Aβ in the walls of intracranial arteries and the very low levels of tracer and Aβ in the walls of the carotid artery in the neck [123, 131] together with the presence of lymph nodes within the carotid sheath just below the base of the skull in humans strongly suggest that ISF and solutes leave artery walls in the neck to drain to adjacent cervical lymph nodes [30]. Quantitative studies of lymphatic drainage from the brain have shown that the speed of drainage is comparable to lymphatic drainage from other organs [131]. The quantity and volume of fluorescent tracers injected to outline the basement membrane pathways for drainage of ISF from grey matter [24] are too small to be detected in the cervical lymph nodes. However, there is further evidence of drainage of solutes from the brain to cervical lymph nodes from transgenic mice that overexpress amyloid precursor protein and Aβ. In these mice, the amount of Aβ in the cervical lymph nodes reflects the degree of production of mutant Aβ in the brain parenchyma [105].

### Impairment of intramural perivascular drainage

There is impairment in the clearance of soluble tracers along basement membranes with increasing age, hyperlipidaemia, and with possession of Apolipoprotein ε4 genotype (*APOE4*). Impairment is associated with age-related changes in the artery walls and biochemical changes in vascular basement membranes [52–55]. It is also possible to block the perivascular drainage pathways along arterial basement membranes acutely as demonstrated by the impairment of drainage when Immune complexes lodge in arterial basement membranes [27]. Epigenetic modifications of the proteins that constitute the extracellular matrix appear to have a role in the properties of the cerebrovascular basement membranes [83] and may be involved in the impaired drainage of fluid and solutes from the brain associated with hyperlipidaemia [53].

Accumulation of fluid in the cerebral white matter with age and Alzheimer’s disease in humans is detected as white matter hyperintensities (WMH) by MRI. One of the factors involved in the aetiology of WMH appears to be impaired drainage of fluid along intramural perivascular pathways in cerebral arteries affected by cerebral amyloid angiopathy [140]. Similarly, the occurrence of amyloid-related imaging abnormalities (ARIA) in the white matter of patients treated with Aβ immunotherapy for Alzheimer’s disease may be due to the increase in accumulation of Aβ in intramural perivascular drainage pathways as CAA, following the release of Aβ from the brain parenchyma [20].

### Motive force for intramural perivascular drainage along cerebrovascular basement membranes

The observation that perivascular transport only occurs in living animals and ceases immediately after cardiac arrest suggests that pulsations in artery walls may generate the motive force for the transport of ISF and solutes out of the brain [24]. Furthermore, mathematical models indicate that perivascular transport of ISF and solutes may be driven by the contrary (reflection) waves that follow each pulse wave [119]. The contrary wave travels in the reverse direction to the major pulse wave and in the reverse direction to the flow of blood. If the contrary wave does act as the motive force for the drainage of ISF and solutes, then the model dictates that some form of valve-like action is required to prevent reflux during the passage of the major pulse wave along the vessel wall [119]. As the route of drainage is within basement membranes [24], it is possible that the valve-like action results from changes in the orientation of the molecules within the basement membranes [122]. As vessels age, they become arteriosclerotic, stiff, and less elastic, particularly in humans, and such stiffening may interfere with perivascular drainage of ISF and soluble metabolites in elderly individuals [59, 139].

Ischaemic stroke as well as amyloid accumulation results in a failure of perivascular clearance of solutes in the affected hemisphere [10]. Cerebral hypoperfusion in a mouse model of Alzheimer’s disease leads to accelerated accumulation of Aβ in the walls of leptomeningeal vessels and this can be reversed by phosphodiesterase inhibitor and vasodilator Cilostazol [102]. Thus, experimental evidence again suggests that the interplay between the strength of pulsations and vasomotion may be key to the efficient perivascular clearance along basement membranes.

### Interrelationships between CSF and ISF: convective tracer influx/glymphatic system

CSF has been shown to circulate through the brain and mix with ISF. When horseradish peroxidase or fluorescent tracers are infused into the cerebral CSF, they enter the surface of the brain along the outer aspects of penetrating arteries and mix with ISF in the ECS of the brain parenchyma [62, 113]. Entry of tracers in the CSF into the brain is along basement membranes between the outer pial coating of the artery and glia limitans [91] (Fig. 4) and appears to be driven by arterial pulsations [62, 113]. Mixing of CSF with ISF is dependent upon the presence of astrocytic aquaporin 4 [62]. Clearance of tracers that enter the brain from the CSF appears to be via paravenous flow either into the CSF or possibly to cervical lymph nodes [62]. The immunological significance of the convective tracer influx/glymphatic system [62, 113] is unclear. Fluid and tissue metabolites in this system appear to drain back into the CSF and may reach lymph nodes via the CSF.

The convective tracer influx/glymphatic system is largely separate from the rapid, direct drainage of ISF from the brain parenchyma along the basement membranes in the walls of cerebral arteries (intramural perivascular drainage) to cervical lymph nodes (Fig. 4). The exact relationship between the two systems, however, requires further investigation.

## Pathways for the migration of antigen-presenting cells from the CNS to regional lymph nodes

Dependent on dynamic local tissue conditions, the fates of leukocyte subsets migrating into the CNS parenchyma are fourfold: (1) transformation into another functional subset, e.g., adoptively transferred Th17 cells changing into Th1 or IFN-gamma producing cells, or Th1* (IFN-gamma and IL-17 producing cells) or even Th2 cells [23], (2) eventual demise in situ and uptake by phagocytic cells, (3) long-term residence, that is presumably rare, and (4) emigration to secondary lymphoid organs. Figures 1 and 2, together with the preceding sections in this review summarize current views on cell migration pathways out of the brain.

The proportional contributions of the different pathways out of the brain and spinal cord are poorly understood. For instance, it is not fully understood why there is such a preponderance of drainage to deep cervical lymph nodes compared to superficial cervical lymph nodes. It is also useful to draw attention here to the fact that cells carrying CNS components can also gain access to the spleen, through the blood. For example, a significant number of cells containing proteolipid protein and a few cells bearing myelin basic protein are present in the red pulp and around vessels within the spleens of marmoset monkeys (*Callithrix jacchus*) suffering from experimental autoimmune encephalomyelitis (EAE), an animal model for multiple sclerosis. In contrast, the spleens of adjuvant controls and untreated monkeys were almost completely devoid of cells containing myelin basic protein and proteolipid protein [36].

Much more is known about lymphatic drainage from the brain than from the spinal cord. Despite the fact that rodent EAE, produced by many induction protocols, is to a large extent a spinal cord disease, drainage to local lymph nodes remains largely unexplored. In the mouse, it seems obvious to assume that the lumbar lymph nodes drain the spinal cord. In humans, with 500–700 lymph nodes in total, and many of them potentially receiving drainage from the spinal cord, the identity of the draining nodes appears to be less clear. Using a chronic-relapsing EAE model in Biozzi ABH mice, van Zwam et al. [135] surgically removed a total of thirteen lymph nodes draining the CNS: eight superficial cervical lymph nodes (also called submandibular, facial, and jugular lymph nodes), two deep cervical lymph nodes (also called internal jugular lymph nodes), and the three lumbar lymph nodes (also called caudal of sacral or para-aortic lymph nodes). This led to a reduced severity of relapses and reduced spinal cord pathology. Furtado et al. [44] had similar results upon extirpation of deep and superficial cervical lymph nodes in a transgenic spontaneous EAE model, as did Phillips et al. [106] in a cryolesion-enhanced model of cerebral EAE. These independent studies support the notion that lymph nodes draining the CNS contribute to disease pathology.

## Tolerance

Although contributions of cervical lymph nodes to detrimental immunity in multiple sclerosis (MS) and EAE may seem rather obvious, it is important to point out that the cervical lymph nodes clearly can also mediate the induction of tolerance [88, 143]. For example, the injection of myelin basic protein into the CSF induces tolerance and prevents the subsequent induction of EAE [49]. Intranasal administration of antigen also drives tolerance through superficial cervical and internal jugular lymph nodes [143].

It is entirely possible that the induction of tolerance versus generation of (auto)immune responses in lymph nodes draining the CNS occurs sequentially or even in parallel. We argue that this is played out at the level of the individual T cell interacting with an individual APC dependent on the reciprocal quantitative and qualitative spectrum of signal 1 (MHC-peptide), signal 2 (panel of costimulatory and co-inhibitory receptors), and signal 3 (spectrum of soluble cytokines). As reviewed by Card et al. [28], lymphatic endothelium can play active roles in regulating host immunity. Secretion of immunosuppressive factors limits T-cell function and DC maturation. In addition, endothelial cells can directly drive T-cell tolerance by antigen presentation in the context of co-inhibitory ligands. Finally, lymphatic endothelium controls delivery of antigens in the lymph by scavenging and regulation of transendothelial transport. In the draining lymph node, immature DC in and lymph node stromal cells can present self-antigen to T cells in a tolerogenic fashion.

Recent studies claim that in experimental stroke, autoimmunity does develop after massive release of CNS antigens, [133]. This follows up on studies by the same group demonstrating brain antigens in cervical lymph nodes of patients with stroke; there is some correlation between antigen load, type, and clinical outcome [108]. Very little evidence of MS or encephalitis has emerged following strokes,

### Events in lymph nodes draining the CNS

Very recently, in back-to-back studies, the next generation sequencing was used to assess B-cell receptor repertoire in MS in peripheral blood versus CSF [104] and in brain versus cervical lymph nodes [82, 128]. Stern and colleagues [128] found B-cell populations with sequences closely resembling the germline, dubbed founder events, which were highly represented in both the CNS and cervical lymph nodes. These are likely to undergo additional rounds of affinity maturation. The first antigen-dependent maturation of B cells would occur in the lymph nodes, as expected, but the lineage trees of the mutated clones suggest that there is traffic in both directions and that affinity maturation can also take place in CNS-related compartments, such as tertiary lymphoid structures in the meninges. The data collectively suggest that there is bidirectional traffic between the CNS and cervical lymph nodes.

The surge of interest in contributions of the gut microbiome to risk and progression of MS has direct bearing on the cervical lymph nodes, with two prime examples from mouse EAE models. First, in a transgenic spontaneous EAE model driven by CD4^+^ T cells against MOG92-106, in which germfree conditions abolish disease induction, the cervical lymph nodes feature an ongoing MOG-specific germinal centre reaction [18]. Second, work from Lloyd and Dennis Kasper on how polysaccharide A from *Bacteroides fragilis* protects against EAE demonstrates increased numbers of regulatory T cells in cervical lymph nodes [98]. These studies underscore the importance of cervical lymph nodes for both T-cell and B-cell responses.

## Efferent pathways between lymph nodes and the CNS

### Activation of T cells that seek the CNS

The deep cervical and lumbar lymph nodes have been shown to a function as lymph nodes that drain the CNS [34]. Thus, effector T and B cells specifically targeting CNS antigens could well be activated in cervical and lumbar lymph nodes and might be imprinted there with CNS-specific-trafficking programs similar to those described above for lymph nodes draining skin and gut, respectively.

Our current knowledge of the anatomical routes and molecular mechanisms involved in the trafficking of immune cells from secondary immune organs into the CNS is mostly derived from studies in murine EAE, an animal model for MS that is focussed mainly on the spinal cord [39]. EAE can be induced by subcutaneous immunization of susceptible mice or rats with neuroantigens emulsified in adjuvants. In this case, autoreactive effector T cells that are normal constituents of the immune repertoire [103, 120] are activated in lymph nodes draining the skin. Alternatively, EAE can be induced by adoptive transfer; this entails the injection into susceptible recipients of neuroantigen-specific CD4^+^ T-cell blasts that have been activated in vitro. Previous studies have provided direct evidence for the migration of low numbers of encephalitogenic T-cell blast across the BBB in the spinal cord within several hours after transfer [56, 134]. However, recent studies performed in a Lewis rat model of EAE have shown that the majority of intravenously inoculated encephalitogenic T-cell blasts do not directly cross CNS microvessels, but rather accumulate in the lung [43, 100]. In the lung and its draining lymph nodes, the T-cell blasts were found to profoundly reprogram their gene expression profile, i.e., they upregulated a migratory program involving adhesion molecules, motility factors, and chemokine receptors, but down-regulated their activation program [100]. These newly-gained migratory properties enabled that these T cells to efficiently cross the BBB [13, 68]. Interestingly, encephalitogenic T cells were also shown to persist as long-lived memory cells within the lung tissue, where they could be stimulated to gain the competence to enter the CNS and trigger autoimmune disease within the CNS. Epidemiological studies suggest that infections of the respiratory tract play an important role in triggering relapses in MS, i.e., relapses in MS regularly follow inflammation of the respiratory tract [124]. Therefore, it is quite conceivable that stimulatory environmental factors might directly elicit a pathogenic response of autoimmune T cells within the lung. These studies indicate that, in addition to the cervical lymph nodes and the gut [18], the lung can also serve as a location, where autoreactive T cells potentially become re-activated.

### The three potential routes of entry for T cells into the CNS

**Fig. 5:**
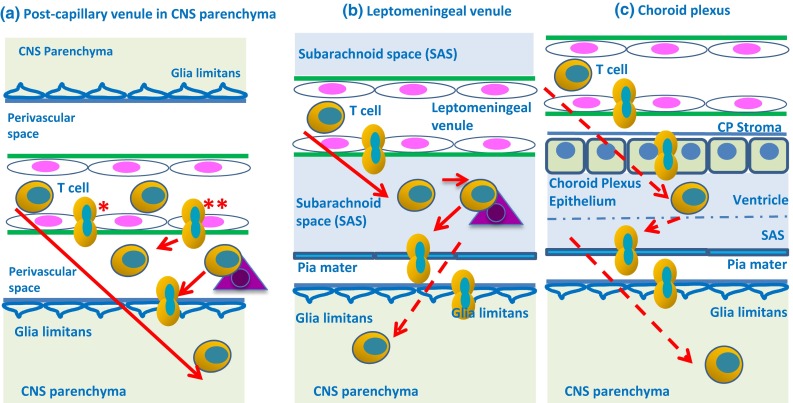
Three potential routes for entry of T-cells into the CNS. **a** Encephalitogenic T cells enter the CNS parenchyma by a first step, passing between (*) or through (**) the BBB endothelial cells of post-capillary venules into the perivascular space, where they need to see their cognate antigen on perivascular APC (*triangle*) before they penetrate the glia limitans as a second step (for details, see text and Fig. 6). **b** In EAE, T cells enter the CSF from leptomeningeal venules, where they need to see their cognate antigen on leptomeningeal macrophages (*triangle*) and might penetrate the pia mater and glia limitans to enter the CNS parenchyma; this route is less certain in MS (see text). **c** T cells pass from blood vessels into the stroma of the choroid plexus and may then penetrate the choroid plexus epithelium to enter the ventricular CSF. They then pass into the subarachnoid space (SAS) and may penetrate the CNS parenchyma by passing through the pia mater and the glia limitans in EAE; this route is less certain in MS (see text). *Solid lines* direct experimental evidence is available for this route; *interrupted* (*dashed*) *lines* indirect experimental evidence is available

The few studies that have directly addressed the early migratory steps of T cells into the CNS have mostly been performed in the framework of EAE and have suggested three potential routes of entry for encephalitogenic T cells into the CNS (reviewed in [39]) (Fig. 5).

1. Live cell-imaging studies have provided evidence for the migration of encephalitogenic CD4^+^ Th 1 cells across leptomeningeal vessels associated with the spinal cord and the brain [13, 107, 134]. Migration is achieved by engaging constitutively expressed VCAM-1 on the BBB endothelium with activated α4β1-integrins expressed on the surface of the activated CD4^+^ Th1 cells [107, 134] (Fig. 5a).
2. Encephalitogenic T cells have also been shown to directly extravasate via the leptomeningeal microvessels into the subarachnoid space [13, 118] (Fig. 5b).
3. Encephalitogenic CD4^+^ Th17 cells that specifically express the chemokine receptor CCR6 would appear to initiate EAE by crossing the blood–CSF barrier in the choroid plexus by engaging CCL20, which is produced by choroid plexus epithelial cells, but not by endothelial cells of the BBB [112]. With the ventricular CSF, the CD4^+^ Th17 cells seem to travel to the leptomeningeal (subarachnoid) spaces, where they accumulate and trigger neuroinflammation [111, 112] (Fig. 5c).

**Fig. 6:**
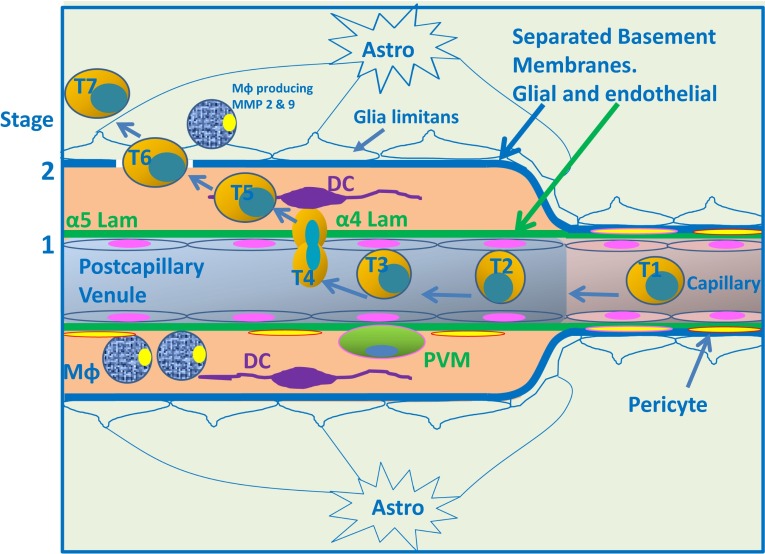
Entry of encephalitogenic T cells directly into the CNS from the blood. A post-capillary venule in an inflamed brain showing the passage of T cells from the lumen into the CNS parenchyma in a two-step process. *Step 1* as a T cell passes from the capillary (T1) to the post-capillary venule, it rolls along the surface of the endothelium (T2) and is arrested by receptors on the T cell and endothelium (T3) (see text for details). The T cell leaves the lumen by diapedesis across the endothelial barrier (T4) and passes through the endothelial basement membrane at sites containing α4 laminin. Areas of basement membrane containing α5 laminin do not allow such diapedesis. The T cell then enters the perivenular space (T5) formed by separation of elements of the glial-endothelial basement membrane (endothelial component:* green*; glial component: *blue*). At this point (T5), there is a step of “re-activation” by recognition of cognate Ag in the perivascular space. As this step also occurs in the absence of neuroinflammation, this holds true for immune surveillance. Infiltrating T cells can recognize their cognate antigen on rare perivascular dendritic cells (DC) and on monocyte-macrophages (Mφ). *Step 2* of traffic from blood to CNS involves penetration of the basement membrane of the glia limitans (T6). Recognition of antigen leads to local T-cell activation and upregulation of further trafficking cues on the BBB endothelium allowing for entry of additional immune cells, including bone marrow-derived macrophages and DCs, into the perivascular space. This leads to local expression of matrixmetalloproteinases (MMP) 2 and 9 produced by macrophages (Mφ) and to cleaving of extracellular matrix receptors of astrocytic end feet that allow inflammatory cells to enter the CNS parenchyma across the glia limitans (T7)

All three routes of entry for T cells share a peculiarity that the cell invasion of the CNS parenchyma requires breaching of a second barrier, and thus, in total, the sequential breaching of two different tissue barriers (Fig. 5). As a first step, the effector T cells must migrate across any of the outer barriers, such as the endothelium of post-capillary venules (Fig. 6). As a second step, effector T cells must progress across the glia limitans that forms the layer of tissue that surrounds microvessels in the CNS parenchyma and forms the surface of CNS apposed to the leptomeninges and the subarachnoid space. Clinical EAE starts when inflammatory cells breach the glia limitans bordering the perivascular spaces or the surface of the brain or spinal cord. The molecular mechanisms mediating the migration across these two different barriers have not yet been clarified in all their details, but they do include distinct molecular cues.

In healthy individuals, it is mostly central memory T cells and B cells and low numbers of innate immune cells that are detected in the CSF [7, 46, 71, 111]. This further underscores the ability of activated T and B cells to breach the outer brain barriers in the absence of neuroinflammation and perform immunosurveillance of the CNS by entering the CSF spaces.

Irrespective of having breached the endothelial BBB or the epithelial blood–CSF barrier, T cells will encounter APC that are resident in the CNS and strategically localized in perivenular spaces and in CSF drained spaces right behind these brain barriers (summarized in [39, 111]). While the perivascular spaces around post-capillary venules harbour rare DCs [48], large numbers of macrophages are found in the subarachnoid spaces [13]. In addition, cells that express MHC class II (Kolmer or epiplexus cells) adhere as APC to the apical aspect of the choroid plexus epithelial cells that form the blood–CSF barrier [78]. In the framework of EAE, it has been shown that the recognition of their cognate antigen on these APC is prerequisite for the subsequent migration of CD4^+^ T cells across the glia limitans into the CNS parenchyma to cause clinical EAE [13]. Direct evidence for this scenario has been derived from live cell-imaging studies of the spinal cord leptomeningeal spaces. The leptomeningeal vessels are embedded in a three-dimensional extracellular matrix network consisting mainly of collagen fibres. T cells having crossed the blood–leptomeningeal barrier will encounter numerous macrophages distributed within the leptomeningeal 3D milieu. These cells actively scan their environment with their cell processes [13]. Effector T cells migrating within the leptomeningeal environment are in regular contact with these cells that constitutively express MHC class II molecules on their membrane surfaces and thus are able to present myelin antigens to the pathogenic effector T cells [13, 70, 99]. In fact, live cell-imaging studies with activation-driven biosensors are able to demonstrate that T cells recognize their cognate antigen on these APC [80, 92] (the cognate antigen is the antigen that the T cell first encountered and which bound to its receptor and resulted in the cell proliferation and differentiation that generated the population of T cells sensitized to that antigen). The resulting antigen-specific activation of the T cells is not only of relevance for the adherence of the T cells to the leptomeningeal milieu [118], but also for the consecutive expression of pro-inflammatory cytokine by the effector T cells that constitutes a crucial signal for the initiation of the parenchymal invasion of immune cells and the clinical autoimmune process [67]. The local T-cell activation triggers inflammatory events that lead to the upregulation of additional adhesion molecules and chemokines on endothelium at the BBB and epithelium at the blood–CSF barrier, thus, allowing the recruitment of additional circulating immune cells, including DC and monocytes, across the brain barriers. Lack of T-cell activation by leptomeningeal macrophages will lead to T-cell detachment and their release into the CSF which flows largely driven by respiration rather than the cardiac cycle [37, 118] in this space.

### Entry of T cells across inflamed brain barriers

Live cell-imaging studies have shown that the extravasation of circulating T cells across the inflamed BBB (activated by pro-inflammatory cytokines with high expression of ICAM-1, VCAM-1, and de-novo expression of P-selectin and other adhesion molecules) is mediated by a multi-step cascade starting with P-selectin glycoprotein-1 (PSGL-1)-mediated T cell rolling on endothelial P-selectin [116] (Fig. 6). As T cells in the blood slow down, they recognize, as yet unidentified, ligands on endothelial cells that bind to G-protein-coupled receptors on the T cells inducing “inside-out” activation of constitutively expressed integrins on the T-cell surface. Integrins are heterodimers constitutively expressed on circulating immune cells in a “bend down conformation” that does not allow engagement with their ligands. Binding of a chemokine to its chemokine receptor triggers a downstream signalling cascade in the immune cell that eventually leads to what is referred to “inside-out” activation of the integrins which then engage talin and kindling; with this, the integrins on the outside of the cell change their conformation into an upright position—now, the integrin can bind the ligand on the endothelium. In mouse models of EAE, α4β1-integrin (VLA-4) mediates T-cell arrest on the inflamed BBB by interaction with endothelial VCAM-1. Subsequent polarization and extended crawling of the T cells against the direction of flow have been shown in vitro to be mediated by LFA-1 interaction with endothelial ICAM-1 and ICAM-2 as in other vascular beds [1].

Crawling of encephalitogenic T cells against the direction of blood flow on the luminal side of spinal cord meningeal vessels in vivo has first been observed during the initiation phase of EAE in a Lewis rat EAE model [13]. In this case, T-cell adhesion to the endothelium and subsequent crawling was significantly reduced by blocking the function of α4β1-integrins, but not LFA-1 [13]. In addition, it has been proposed that the other adhesion molecules, such as ninjurin-1, ALCAM, and MCAM, participate in T-cell migration across the BBB [61]. A recent in vitro study showed that extensive crawling of encephalitogenic T cells against the blood flow is a unique characteristic when low levels of ICAM-1 are expressed on the BBB allowing the T cells to find sites for paracellular diapedesis [1]. Inflammatory stimuli increased the cell surface levels of ICAM-1 on the BBB endothelium, which abrogated extended T-cell crawling and allowed the T cells to cross the BBB via transcellular routes.

### Role of α4-integrins

Functional inhibition of α4-integrins completely abrogates T-cell entry into the CNS and thus inhibits clinical EAE. This contrasts with blocking P-selectin/PSGL-1 (rolling) and blocking LFA-1/ICAM-1/ICAM-2 (arrest) interactions, which fail to inhibit or only partially inhibit T-cell migration across the BBB. Similar mechanisms for trafficking across the BBB have also been shown for myeloid cells, including DC; this applies especially to the predominant role for α4-integrins [42, 63]. These findings have been translated to clinical use by treating relapsing-remitting MS with the humanized anti-α4-integrin antibody natalizumab [127].

### Role of the glia limitans

As pointed out above, an important observation made in the EAE model is that as long as infiltrating T cells, and other inflammatory cells, reside in the perivascular spaces of post-capillary venules, and in the subarachnoid spaces, the animals do not develop any clinical signs of disease. Clinical EAE does require immune cells to traffic across the glia limitans. This next step involves TNF-α which triggers expression of matrixmetalloproteinase-2 (MMP-2) and MMP-9 in perivascular and leptomeningeal myeloid cells leading to the cleavage of astrocyte dystroglycan which anchors the astrocyte foot processes to the parenchymal basement membrane of the glia limitans [4]. At the same time, the scavenger receptor CXCR7 is upregulated on the inflamed BBB endothelium leading to downregulation in the levels of the chemokine CXCL12 in the perivascular spaces which further facilitates mobilization of CXCR4^+^ T cells from the perivascular space into the CNS parenchyma [31]. In the CSF, CXCL12 acts as a homeostatic chemokine that maintains the expression of its ligand CXCR4 by immune cells, keeping them in the subarachnoid space; this may be relevant for immune surveillance.

In humans, the glia limitans on the surface of the CNS is much thicker than in rodents and consists of multiple layers of astrocyte processes [5]. Although lymphocytes and APC readily penetrate the delicate glia limitans around post-capillary venules in the human brain, the penetration of inflammatory cells through the thicker glia limitans on the surface of the brain and spinal cord is less certain. For example, plaques of demyelination on the surface of the cerebral cortex in MS may be associated with inflammatory cells in the overlying subarachnoid space [57]. However, the penetration of such cells across the glia limitans is not seen. The apposition of inflammatory cells in the CSF and demyelination in the cerebral cortex suggests that the diffusion of soluble agents may either pass from CSF into brain [57], or agents, such as cytokines, may defuse from the brain into the CSF and attract inflammatory cells to the subarachnoid space [8].

### Two-step system for migration of immune cells into the CNS

Taken together, observations of immune cell migration into the CNS that are mainly derived from the EAE model have shown that the migration of lymphocytes into the CNS is tightly controlled by a system of at least two steps with an outer step at the level of the BBB and the blood–CSF barrier and an inner step at the level of the glia limitans (Fig. 6). The perivascular space or the subarachnoid space between these steps serve as checkpoints, in which the molecular keys required for immune cells to breach the glia limitans and to enter the CNS parenchyma are dependent upon local recognition of CNS antigens by the invading T cells [38].

### CD8 T-cell, B cell, and myeloid cell migration into the CNS

Although CD8 T cells contribute to immune surveillance of the CNS [22] and are key players in neuroinflammation [117], little is known about the cellular and molecular mechanisms involved in the migration of CD8 T cells into the CNS. Interaction between PSGL-1 and P-selectin has been shown to contribute to the recruitment of human CD8 T cells in leptomeningeal brain vessels [14]. Recent development of mouse models of CNS autoimmunity driven by CD8 T cells has led to the identification of the first molecular mechanisms involved in cytotoxic CD8 T-cell migration into the CNS and has shown that these mechanisms are distinct from those used by CD4^+^ T cells. Although α4β1-integrin is essential for CD8 T-cell entry into the CNS in vivo, the vascular ligand engaged was identified as junctional adhesion molecule-B (JAM-B) rather then VCAM-1 used by CD4^+^ T cells [84]. A recent side by side comparison of CD8 versus CD4 T-cell migration across an in vitro model of the BBB further substantiated that molecular mechanisms mediating the multistep extravasation of CD8^+^ T cells across the BBB are distinguishable from those involved for CD4^+^ T cells [114] and thus need further investigation.

Even less is known about the molecular mechanisms guiding B cells and myeloid cells into the CNS during immune surveillance or neuroinflammation. Although α4β1-integrins contribute to B cell and myeloid cell entries into the CNS [63, 77], the precise entry routes and molecular mechanisms involved in their multi-step recruitment across the brain barriers remain to be explored.

## Relationships between lymphatic drainage of the brain and neurological disease

### EAE and multiple sclerosis

A number of experiments using different models of EAE suggest the involvement of cervical or lumbar lymph nodes in neuroimmunological disease. Excision of the CNS-draining lymph nodes in chronic-relapsing EAE reduced and delayed the burden of relapse and EAE pathology within the spinal cord. This finding suggests that specific responses to CNS antigens are initiated within lymph nodes that drain the CNS [135]. Further evidence comes from the cryolesion-enhanced form of cerebral EAE in which the removal of deep cervical lymph nodes during the incubation period of EAE and at the same time as the cerebral cryolesion significantly reduces the burden of EAE in the cerebral hemispheres [106]. Transfer of lymphocytes from animals with cryolesion-EAE resulted in EAE lesions that were predominantly in the cerebral hemispheres [73]. These results suggest that lymphocytes from donors with cryolesion-EAE target the brain in recipient animals in preference to the spinal cord. This might be due to tissue-specific cues influencing the function of DC in the draining lymph nodes that allows them to imprint a CNS homing program that is different from EAE without a cryolesion. Enhancement and location of autoimmune inflammation in the brain following a focal cortical cryolesion injury initially involve chemokines, such as the macrophage chemoattractants CCL2 (MCP-1) and CCL12 (MCP-5), and the activities of afferent and efferent neuronal connections with the site of damage [129]. By analogy, similar factors may modulate or reactivate autoimmune inflammation in MS [129].

Induction of a CNS-specific inflammatory response is, however, slow. Experimentally induced death of oligodendrocytes in transgenic mouse models rapidly leads to strong activation of microglia-macrophages and accumulation of myelin components in lymph nodes that drain the CNS. However, the resulting infiltration of immune cells into the CNS does not occur until weeks after the insult [79, 132].

In MS, neuronal antigens were present in pro-inflammatory antigen-presenting cells in cervical lymph nodes, whereas the majority of cells containing myelin were anti-inflammatory [136]. This may reflect a different origin of the cells or different drainage mechanisms. Indeed, cells containing neuronal antigens in human cervical lymph nodes did not express the lymph node homing receptor CCR7, whereas cells containing myelin antigens in situ and in vitro did. Nevertheless, cervical lymph nodes from CCR7-deficient mice affected by EAE contained the same amounts of myelin and neuronal antigens as wild-type mice. It was concluded from this study [136] that the type and frequencies of CNS antigens within the cervical lymph nodes are determined by the type and extent of CNS damage. Furthermore, the presence of myelin and neuronal antigens in functionally distinct APC populations within MS cervical lymph nodes suggests that differential immune responses can be evoked.

There is no evidence from clinical experience in MS that cervical lymph nodes are enlarged or reactive, but this does not appear to have been pursued by systematic imaging [40]. However, there is great promise and opportunity in the development of advanced imaging methods both in experimental animals, and in humans. So far, there has been very little effort to image lymph nodes that drain the CNS in MS patients, perhaps understandably since there is no overt evidence for lymph node swelling. Progress in sonography, computed tomography, and magnetic resonance imaging could yield useful insights (reviewed in [75]), facilitated by the fact that cervical lymph nodes are relatively near to the skin. Novel imaging agents for brain compounds should be very informative for imaging the transport of soluble compounds, such as, e.g., myelin fragments, lipids, and proteins, molecules derived from axons and Aβ species. The ultimate aim would be in vivo labelling with probes suitable for clinical use and at a resolution that would allow imaging of single cells and soluble antigens. For instance, Aβ concentrated in basement membrane compartments, or myelin and axonal compounds in lymphatic vessels and in lymph node conduits could be labelled for detection by imaging techniques.

### Cerebral amyloid angiopathy and Alzheimer’s disease

The brain has a unique and somewhat restricted pathway for the elimination of ISF and solutes from its parenchyma to cervical lymph nodes that serves not only an immunological function but is also a pathway for the elimination of soluble metabolites from the brain [25]. This system of elimination fails with age [54], and this appears to be a major factor in the aetiology of CAA and Alzheimer’s disease.

Amyloid-β (Aβ) is a peptide that can exist as soluble, oligomeric, insoluble, and β-pleated sheet fibrillar forms in the extracellular spaces of the brain parenchyma and in the walls of cerebral arteries and capillaries as CAA in the ageing human population and in Alzheimer’s disease. Aβ is derived from amyloid precursor protein and appears to be produced by the majority of cells in the body, including the brain, throughout life. Elimination of Aβ from the brain entails absorption into the blood, degradation by enzymes, such as neprilysin, and drainage, along intramural perivascular routes with ISF to lymph nodes [25]. With age and Alzheimer’s disease, insoluble fibrillary Aβ is deposited in intramural basement membranes of capillaries and arteries of the brain as CAA. Age-related changes in cerebral arteries impair intramural perivascular drainage of ISF [54], and this may be a trigger for the amyloid cascade, loss of homeostasis and propagation of tau protein in the brain in Alzheimer’s disease [139].

## Immune privilege

The original experiments demonstrating immune privilege in the brain showed that skin grafts directly implanted into the brain survived, but were rejected when similar grafts were placed on the skin [86]. However, if grafts extended into the ventricles and thus the CSF, they were rejected [86]. It has also been shown by several groups and more recently directly by live cell imaging that activated T cells can enter the CSF in the leptomeningeal spaces irrespective of their antigen-specificity; this closely resembles the routine immunosurveillance of tissues performed by T cells in organs other than the CNS [118]. These observations emphasise that, although there is immune privilege in the CNS, such privilege does not extend to the ventricles and leptomeningeal spaces that contain CSF.

Except for penetrating injuries of the brain or spinal cord, foreign material, including microorganisms, does not enter the CNS unless they have passed through peripheral tissues, such as lung, gut, skin, or nasal mucosa. Thus, it appears that T-cell-mediated immune responses, such as those involved in the rejection of skin allografts, in the elimination of microorganisms and in autoimmune disorders, may occur in the CNS only if there has been initial immunization by antigen in peripheral tissues. There are also other factors that contribute to the unique interrelationships between the CNS and the immune system. They include barriers to the entry of T cells and other inflammatory cells into the CNS in addition to restricted pathways for APCs to traffic from the CNS parenchyma to lymph nodes; these barriers and pathways have been outlined in the review above.

In summary, the main factors that contribute to Immune Privilege of the CNS are:

1. The afferent intramural perivascular drainage pathway for ISF from CNS parenchyma to regional lymph nodes is restricted to fluid and solutes and does not allow the traffic of APC. The only route for trafficking of APC to regional lymph nodes is by the lymphatic drainage of CSF.
2. There is no free diffusion of plasma filtrates across the blood–brain barrier or across the blood–CSF barrier into the CNS.
3. There is a blood–brain barrier and a blood–CSF barrier for the entry of lymphocytes and other inflammatory cells into the CNS. For lymphocytes, there is a two-step process by which T cells first cross the post-capillary venular endothelium by receptor-mediated mechanisms and then a second step by which T cells cross the glia limitans. Such a restrictive pathway for the entry of T cells and other inflammatory cells is not seen in peripheral tissues. There is also restricted entry for polymorphonuclear leukocytes into the CNS; they are mostly seen in the CSF and in the CNS parenchyma in response to bacterial or fungal infections.
4. In contrast to the skin and other peripheral tissues, the CNS does not harbour resident T cells in the parenchyma, although they reside in ventricular and subarachnoid compartments.
5. There is a lack of constitutive expression of MHC class II and class I in the parenchyma of the CNS which prohibits antigen presentation in the absence of inflammatory stimuli.
6. Microglial cells are a unique population of yolk sac-derived tissue myeloid cells that migrate into the CNS during fetal development; they exhibit different functions from APC in perivascular compartments of the CNS and in the CSF.

## Conclusions

Recent advances in technology have increased our understanding of the relationship of the CNS to the immune system. This knowledge has been translated into the treatment of relapsing-remitting MS with the humanized anti-α4-integrin antibody natalizumab and to understanding perivascular pathways for the elimination of Aβ from the brain and their significance in Alzheimer’s disease, to give but two examples. However, our understanding is not complete. We look forward to further developments in advanced imaging techniques in experimental animals and also in humans to investigate migration of APC from CSF to lymph nodes. Progress in sonography, computed tomography, and MRI could yield useful insights into the physiology of cervical lymph nodes. Novel imaging agents for brain compounds could be developed to image the transport of soluble compounds from the brain along intramural perivascular drainage pathways to lymph nodes. This would aid the investigation of transport of cells and soluble antigens from the brain parenchyma and CSF in neuroimmunological disorders. Evaluation of the age-related failure of elimination of Aβ may lead to therapies to facilitate the elimination of Aβ from the brain in the management and prevention of Alzheimer’s disease.

## Abbreviations

APC: Antigen-presenting cell
BBB: Blood–brain barrier
CAA: Cerebral amyloid angiopathy
CNS: Central nervous system
CSF: Cerebrospinal fluid
DC: Dendritic cell
EAE: Experimental autoimmune encephalomyelitis
ECS: Extracellular space
ISF: Interstitial fluid
MS: Multiple sclerosis
SAS: Subarachnoid space
